# Endoprosthetic reconstructive surgery with medical grade long term implantable silicone in facial asymmetry

**Published:** 2012-12-25

**Authors:** E Lăţcan, CR Popescu

**Affiliations:** * “Prain" Medical Center, Bucharest, Romania; ** ENT&HNS Department, “Colţea" Clinical Hospital, Bucharest, Romania

**Keywords:** endoprosthetic reconstruction, medical grade long-term implantable silicone, congenital malformation

## Abstract

The authors present their experience over a period of 13 years (1998-2011) regarding a cohort of 54 patients.

In an extensive loss of tissues resulted from congenital malformations (maxillary and mandibular malformations, micro stoma), collagenosis (scleroderma, Romberg Syndrome), traffic and work accidents, post surgical (cancer and facial nerve paralyze), when usual surgical procedures fail to establish the normal look of the patient medical grade long-term implantable silicone endoprosthetic reconstruction (rehabilitation) intervenes.

Using a specific technique and materials like long-term implantable silicone grade, the resulted endoprostheses replace and create the aesthetic and a normal anatomy of the specified region, very well tolerated, elastic and non-allergic and with a perfect acceptation from the body all the life.

## Introduction

In an extensive loss of tissues resulted from congenital malformations (maxillary and madibular malformations, microretrognatia, microstoma), collagenosis (scleroderma, Romberg Syndrome), traffic and work accident, post surgical (cancer and facial nerve paralyze), when usual surgical procedures fail to establish the normal look of the patient, silicone endoprosthetic reconstruction (rehabilitation) intervenes. 

 Using a specific technique and materials like silicone grade, the resulted endoprostheses replace and create the aesthetic and a normal anatomy of the specified region, very well tolerated, elastic and non-allergic and with a perfect acceptation from the body all the life. The silicone is a synthetic material, very well accepted and tolerated by the human body, being thermally stabile, lightweight and oxygen transporter. 

There two kinds of silicones:

- Silicone elastomers

 - Implantable silicone [**4**] – there are two commonly used types: long term and short term. 

 Short-term implantable silicone is used up to 29 days or less (suture sleeves, connectors and manifolds).

 Long-term implantable silicone is usually used for 30 days or more (neurostimulators, heart valves and pacemakers).

 Indications: Sadly, in our country, a considerable number of people acquire each year varying facial defects as a result of malignant disease, trauma and congenital deformation.

In similar cases, the age and general medical condition of the patient may also contra-indicate major reconstructive surgery (but not in prosthetic rehabilitation).

 Treacher Collins syndrome (mandibulofacial dysostosis) - also known as Franceschetti syndrome is a rare congenital disorder characterized by craniofacial deformities, [**5**] the auricle is small, malformed or absent, facial anomalies, including macrostomia, micrognathia, malar hypoplasia, conductive hearing loss, underloop zygoma.

 Pery-Romberg syndrome is characterized by slowly progressive hemi facial atrophy of skin and soft tissues, usually on the left side. It is more common in females than in males. It is also accompanied by neurological abnormalities including seizures and episodes of severe facial pain (trigeminal neuralgia). Muscles in the face may atrophy and there may be bone loss in the facial bones. Reconstructive surgery is needed to repair wasted tissue.

 Reconstructive surgery with endoprostheses consists in two stages:

 a. making of the endoprotheses – by the prosthetic doctor; 

 b. surgery technique of insertion and fixation of the prostheses by collaboration with a plastic, ENT, ophthalmology, oncology, neurosurgeon and general surgeons.

 The presence of the reconstruction technique [**1**] before, during and after the surgery procedure means: 

 - taking of the facial impression [**2**],

- wax sculpting,

 - plaster mould construction, 

- silicone selection [**3**],

- silicone endoprostheses cure, 

- trimming and finishing, 

- sterilization of final endoprostheses.

 Surgery technique:

 - creating the space under the skin for the insertion of endoprostheses;

 -the insertion and fixation of the prostheses;

-control of homeostasis; 

 - dressing.

 Materials:

 - impression alginate and silicone;

 - medical grade long-term implantable silicone + activator;

 Surgical instruments: specific of a dental technique and general surgery;

 Complications:

 - local irritation; 

 - suprainfection;

 - rejection of the silicone endoprosthesis, for immunologic reason.

 Congenital malformation

 Case 1

**Fig. 1:**
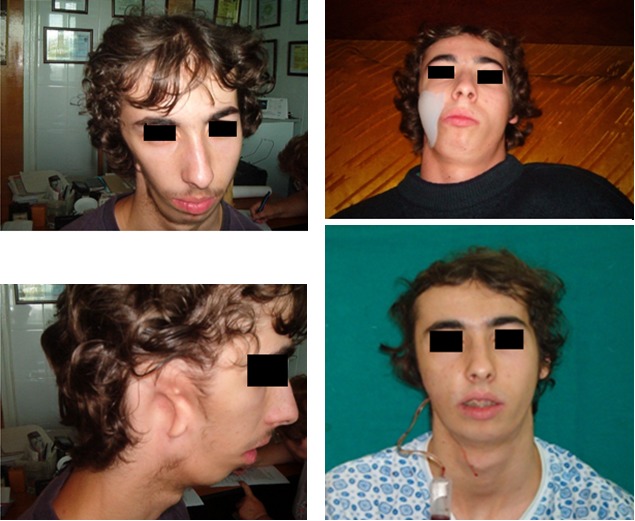
L.M. 22 years old, Francescheti syndrome with ear congenital malformation and hemi facial asymmetry. Using implantable silicon grade endoprostheses inserted in the right cheek obtaining a normal look of the patient

 Romberg syndrome

 Case 2

**Fig. 2:**
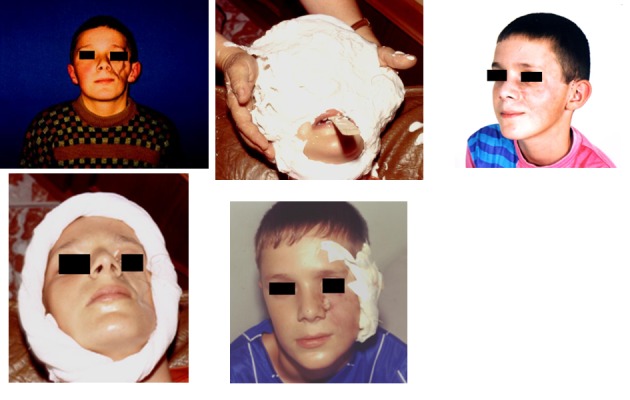
M.A., 10 years old, Timisoara. Facial rehabilitation using endoprosthesis made out of implantable silicone grade, inserted under the skin by plastic surgery.

Case 3

**Fig. 3:**
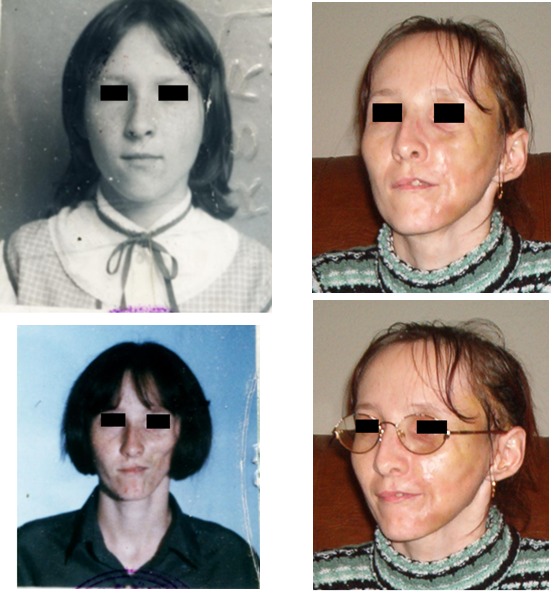
S. L., 27 years old, Deva - Hunedoara. An implantable silicone grade endoprosthesis was made and fixed

 Accident

 Case 4

**Fig. 4:**
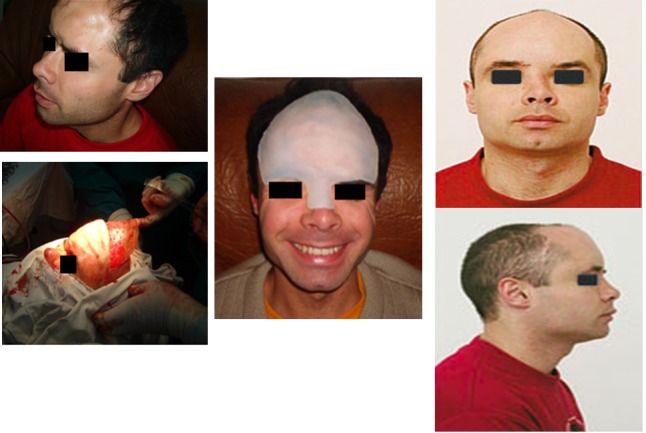
V. S. T.L., 28 years old, Sfantu Gheorghe, suffers a sledding accident (hitting with violence the skull in the trunk of a Christmas tree) in Poiana Brasov, with subsidence of the region and the base front nose, hyperthelorism and 3 months profound coma. After repeated unsuccessful attempts in terms of aesthetic reconstruction of the frontal region acrylate neurosurgery and ENT to the nose with iliac crest bone graft, the patient has rebuilt the front region and nasal pyramid after nearly 10 years with a silicone endoprosthesis subcutaneously implantable introduced by surgery under general anesthesia.

 Case 5

**Fig. 5:**
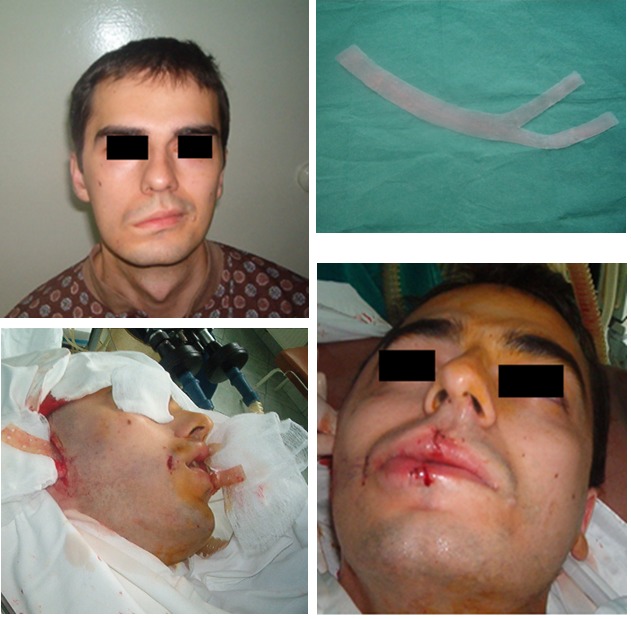
G.B., 26 years old, with facial asymmetry after right acoustic neuromata surgery, 2 cm silicone grade band was made, introduced and fixed under temporal and lips orbicular muscle with good aesthetical results after 6 months.

 Bilateral sclerodermy

 Case 6

**Fig. 6:**
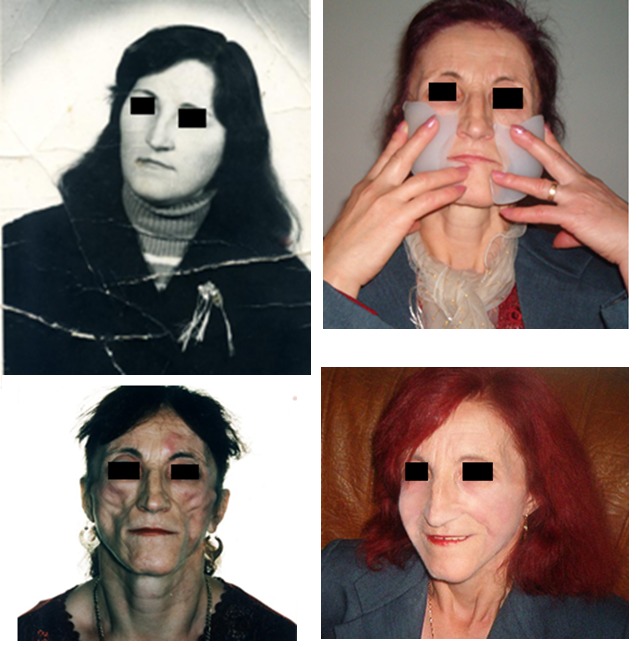
S.V., 48 years old, Timisoara, after unsuccessful medical treatment; endoprosthetic reconstruction with implantable silicone grade was made

 Perinatal accidents

 Case 7

**Fig. 7:**
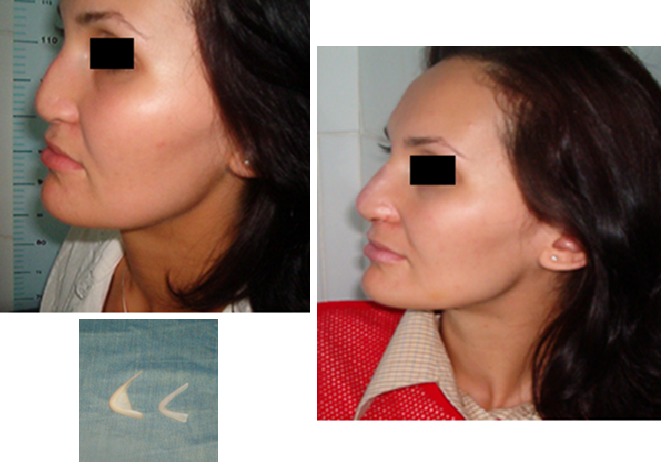
M.L. 29 years old, Teleorman, at birth, with obstetrical methods pyramid nasal is squeezed. Numerous surgical tries (bone, cartilage) failed; surgical reconstruction with silicon

 Case 8

**Fig. 8:**
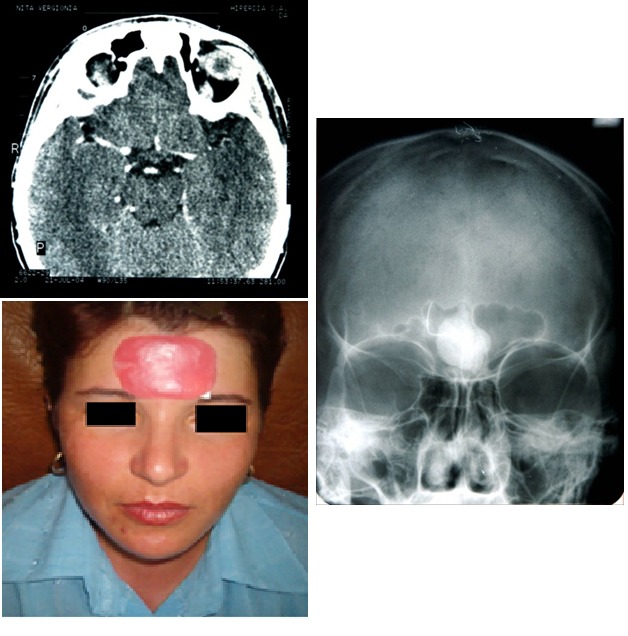
N.V., 34 years old, implantable silicone grade endoprosthetic surgical reconstruction of past wall of frontal sinus after extraction of benign tumor: osteoma

## Conclusions

Prosthetic reconstructive surgery using endoprosthesis from medical grade long-term implantable silicone:

 - personal method;

 - it is a modern method in an extensive loss of substance when common surgery is unsuccessful;

 - with psychological implications in socialization and giving better quality of patient’s life.

 Medical grade long-term silicone endoprosthesis:

 - have the ability to remain inside the human body for an extended period of time, more than 29 days, even all life; 

 - aesthetic role, with good integration in craniofacial anatomy;

 - well tolerated all life, elastic, non-allergic and biologically inert and thermally stabile, oxygen transport, having a recognized biocompatibility for human implantation.
